# The clinical characteristics and implications of acute kidney injury during induction therapy for acute promyelocytic leukemia

**DOI:** 10.3389/fphar.2025.1540409

**Published:** 2025-02-11

**Authors:** Kai Shen, Chenlu Yang, Jie Huang, Xiao Shuai, Ting Niu, Hongbing Ma

**Affiliations:** Department of hematology, West China Hospital of Sichuan University, Chengdu, China

**Keywords:** acute promyelocytic leukemia, acute kidney injury, renal failure, all-trans retinoic acid, arsenic trioxide, differentiation syndrome, prognosis

## Abstract

**Background:**

Dual induction with all-trans retinoic acid (ATRA) and arsenic trioxide (ATO) has made acute promyelocytic leukemia (APL) a curable disease. However, differentiation syndrome (DS) can be a life-threatening complication of induction therapy. It is considered to result from a severe systemic inflammatory response mediated by increased expression of cytokines, chemokines, and adhesion molecules on differentiating blast cells. The kidney, as a vital organ rich in the capillary endothelium, could be targeted by differentiating blasts in DS. Acute kidney injury (AKI) is a rare but severe consequence of DS secondary to ATRA and ATO induction and can result in renal failure and early mortality. Nevertheless, its clinical characteristics and impact on APL prognosis have yet to be elucidated.

**Objectives:**

The aim of this study was to describe the clinical characteristics of DS-related AKI in patients with APL and its impact on patient prognosis.

**Methods:**

This was a retrospective study from a single center in a real-world setting. APL patients who developed AKI during ATRA- and ATO-based induction were included. The patients’ clinical/laboratory data and outcome information were retrieved from the electronic medical records.

**Results:**

From January 2011 to March 2024, a total of 26 out of 572 (4.5%) APL patients were identified as having AKI during dual induction. Among them, eight patients received continuous renal replacement therapy, and 3/8 patients experienced early death (ED), which was defined as death within 3 months of diagnosis. Among the five non-ED patients, three did not recover from renal function and were still dialysis-dependent during the follow-up. The estimated 2-year overall survival rate for all patients was 42%, and the ED rate was 30.8%. Survival analysis revealed that a greater tumor burden, a rapidly increasing WBC count, worse coagulation parameters, and persistent renal dysfunction were associated with a more adverse prognosis.

**Conclusion:**

AKI is a rare but severe complication of DS in the ATRA + ATO dual-induction era of APL. It is associated with a high ED rate and dismal long-term survival. Some patients develop irreversible renal dysfunction and become dialysis-dependent after leukemia remission. Thus, the management of AKI in APL patients is still a clinical challenge, and a deeper understanding of its pathogenesis, along with multidisciplinary efforts, is needed.

## 1 Introduction

Acute promyelocytic leukemia (APL) is a distinct acute myeloid leukemia (AML) subtype characterized by balanced reciprocal translocation t (15; 17) involving the promyelocytic leukemia (PML) gene on chromosome 15 and the retinoic acid receptor alpha (RARA) gene on chromosome 17 (De Braekeleer et al., 2014). The advent of all-trans retinoic acid (ATRA) and arsenic trioxide (ATO) has revolutionized treatment by inducing the differentiation and apoptosis of leukemic cells, resulting in a cure rate of more than 90% in APL patients (Stahl and Tallman, 2019). However, differentiation syndrome (DS), formerly known as retinoic acid syndrome, is observed in APL patients treated with ATRA and/or ATO (Yilmaz et al., 2021). Approximately one-quarter of APL patients who are receiving induction therapy will develop DS (Fathi et al., 2021). DS is considered a result of a severe systemic inflammatory response mediated by increased expression of cytokines (cytokine storm), chemokines, and adhesion molecules on differentiating blast cells that obtain some functional characteristics of mature neutrophils (Luesink et al., 2009a). Although a “cytokine storm” has been described to coincide with the differentiation of blasts, the underlying etiopathogenic mechanisms of this syndrome remain somewhat unknown.

Acute kidney injury (AKI) is often a hallmark of severe DS and is associated with increased mortality during the induction period of APL. It was present in 46% of the APL patients with severe DS enrolled in the PETHEMA LPA96 and LPA9 trials during induction therapy with the AIDA regimen (Montesinos and Sanz, 2011). Few patients may develop refractory renal failure and require hemodialysis or continuous renal replacement therapy (CRRT). Nevertheless, until now, as a rare complication of ATRA and ATO dual induction, detailed data regarding its clinical characteristics and implications for APL patients with AKI have been limited to case reports only (Flombaum et al., 1996; Tsuchiya et al., 2011; Jen et al., 2024). The renal outcome and its impact on patients’ long-term prognosis have yet to be elucidated.

Thus, we retrospectively reviewed APL patients with DS-related AKI in a single center in a real-world setting. The aim of this study was to analyze the clinical characteristics of DS-related AKI in patients with APL and its impact on patient prognosis.

## 2 Methods

### 2.1 Study design and participants

This was a single-center retrospective study from a tertiary hospital in Chengdu, China. APL patients who were diagnosed with DS-related AKI and treated at West China Hospital of Sichuan University (WCHSCU) from January 2011 to March 2024 were included. The diagnosis of APL was based on cytomorphology and confirmed by the presence of the PML::RARA fusion gene via a molecular assay in accordance with the Chinese guidelines for the diagnosis and treatment of APL (2018) (Chinese Society of Hematology Chinese Medical Doctor Association and Chinese Medical Association Chinese Medical Doctor Association, 2018). Based on the results of the PETHEMA study, DS was diagnosed using at least two of the following clinical features: dyspnea, pulmonary infiltrates on chest radiography, unexplained fever, effusions (pleural or pericardial), acute renal insufficiency shown by serum chemistry, hypotension, weight gain (>5 kg), and edema (De Botton et al., 1998). AKI was diagnosed according to the KDIGO criteria, which were functionally defined as an increase in serum creatinine (sCr) of ≥50% within 7 days or an increase in sCr of ≥26.5 μmol/L within 2 days or oliguria for ≥6 h (Pereira et al., 2017). Pediatric patients younger than 14 years and patients with a documented previous history of chronic kidney disease were excluded. Clinical data, including demographic data, clinical manifestations, laboratory parameters, imaging, and outcome information, were retrieved from the electronic medical records of WCHSCU.

### 2.2 Treatment

All patients received ATRA- and ATO-based induction therapy, following the 2018 Chinese APL guidelines (Chinese Society of Hematology, Chinese Medical Doctor Association, and Chinese Medical Association Chinese Medical Doctor Association, 2018). Owing to the retrospective nature of the study, the induction protocol, including the dosage of ATRA and ATO, could be dynamically adjusted at the discretion of the attending physician on the basis of the individual condition of each patient. In general, for low- or intermediate-risk APL patients, cytoreductive chemotherapy involving daunorubicin, idarubicin, cytarabine, and/or hydroxyurea at various dosages or in different combinations was added upon the diagnosis of DS. High-risk patients with WBC counts greater than 10 × 10^9^/L were treated with ATRA and ATO plus various cytoreductive regimens (Sanz et al., 2000). If DS was suspected or confirmed, dexamethasone was administered at 10–20 mg daily, and ATRA and ATO were reduced or suspended on the basis of the patient’s condition. Consolidation and maintenance were conducted following the 2014 and 2018 Chinese APL guidelines (Chinese Society of Hematology, Chinese Medical Doctor Association, and Chinese Medical Association Chinese Medical Doctor Association, 2018; Shen et al., 2014).

### 2.3 Definition of response and endpoint

Overall survival (OS) was defined as the time from admission to death or the last follow-up. Early death (ED) was defined as death during the first 3 months of admission. The primary endpoint of the study was OS. The secondary endpoint was ED. The persistence of renal dysfunction was defined as sCr over the upper normal limit (UNL), which was 106 μmol/L for male patients and 97 μmol/L for female patients, or a dependence on renal replacement therapy at the endpoint.

### 2.4 Statistical analyses

All the statistical analyses were performed with SPSS version 29 (IBM Corp., Armonk, NY, United States). Continuous variables are expressed as the median (range). Categorical variables are presented as frequencies and percentages. The missing data were handled via a mean/median substitution approach. Comparisons between normally distributed continuous variables were performed via independent sample t-tests. The Mann‒Whitney *U* test was used to compare non-normally distributed variables. Categorical variables were compared via the chi-squared test or Fisher’s exact test. OS was calculated via the Kaplan‒Meier method and compared between each risk group via the log-rank test. The Cox proportional hazard model was used for multivariate analysis. Variables with *p-*values <0.05 in the univariate analysis were included. A two-tailed *p-value* < 0.05 was regarded as statistically significant.

## 3 Results

### 3.1 Baseline characteristics

A total of 26 out of 572 APL patients (4.5%) who were diagnosed with and treated at our center were identified as having AKI. Among them, 15 (57.7%) were male and 14 (53.8%) were high-risk APL patients. The median white blood cell (WBC) count was 13.97 × 10^9^/L (range, 0.53–152.26 × 10^9^/L). The median age was 44.5 years (range, 15–70 years). Thirteen (50%) patients had renal dysfunction before the initiation of induction therapy. The baseline sCr level was 100 μmol/L (range, 32–376 μmol/L). The baseline characteristics are summarized in Table 1.

**TABLE 1:** Baseline clinical characteristics of APL patients with AKI during induction.

Characteristic	Total patients (n = 26)
	N (%)/median (range)
Gender	
Male	15 (57.7)
Female	11 (42.3)
Age (years)	44.5 (15–70)
Duration of symptoms before admission (days)	5 (1–30)
Laboratory parameters at diagnosis	
Hemoglobin (g/L)	69 (28–154)
Platelet count (×10^9^/L)	25 (3–72)
WBC count (×10^9^/L)	13.97 (0.53–152.26)
Peripheral blood blast percentage	0.66 (0–0.98)
PT (s)	15 (11–48)
APTT (s)	27 (23–64)
TT (s)	18 (15–26)
D-dimer (mg/L)	29.94 (4.04–38.00)
Fibrinogen (g/L)	0.93 (0.50–4.35)
LDH (U/L)	462 (152–2,977)
Serum creatinine (μmol/L)	100 (32–376)
Uric acid (mmol/L)	372 (140–611)
Manifestations and complications at diagnosis	
Mucocutaneous bleeding	14 (53.8)
Pulmonary infiltration on CT	11 (42.3)
Intracranial bleeding on CT	2 (7.7)
Gastrointestinal bleeding	6 (23.1)
Respiratory failure	3 (11.5)
Renal dysfunction	13 (50.0)

Abbreviations: WBC, white blood cell; PT, prothrombin time; APTT, activated partial thromboplastin time; TT, thrombin time; LDH, lactate dehydrogenase.

### 3.2 Renal outcome

**FIGURE 1:**
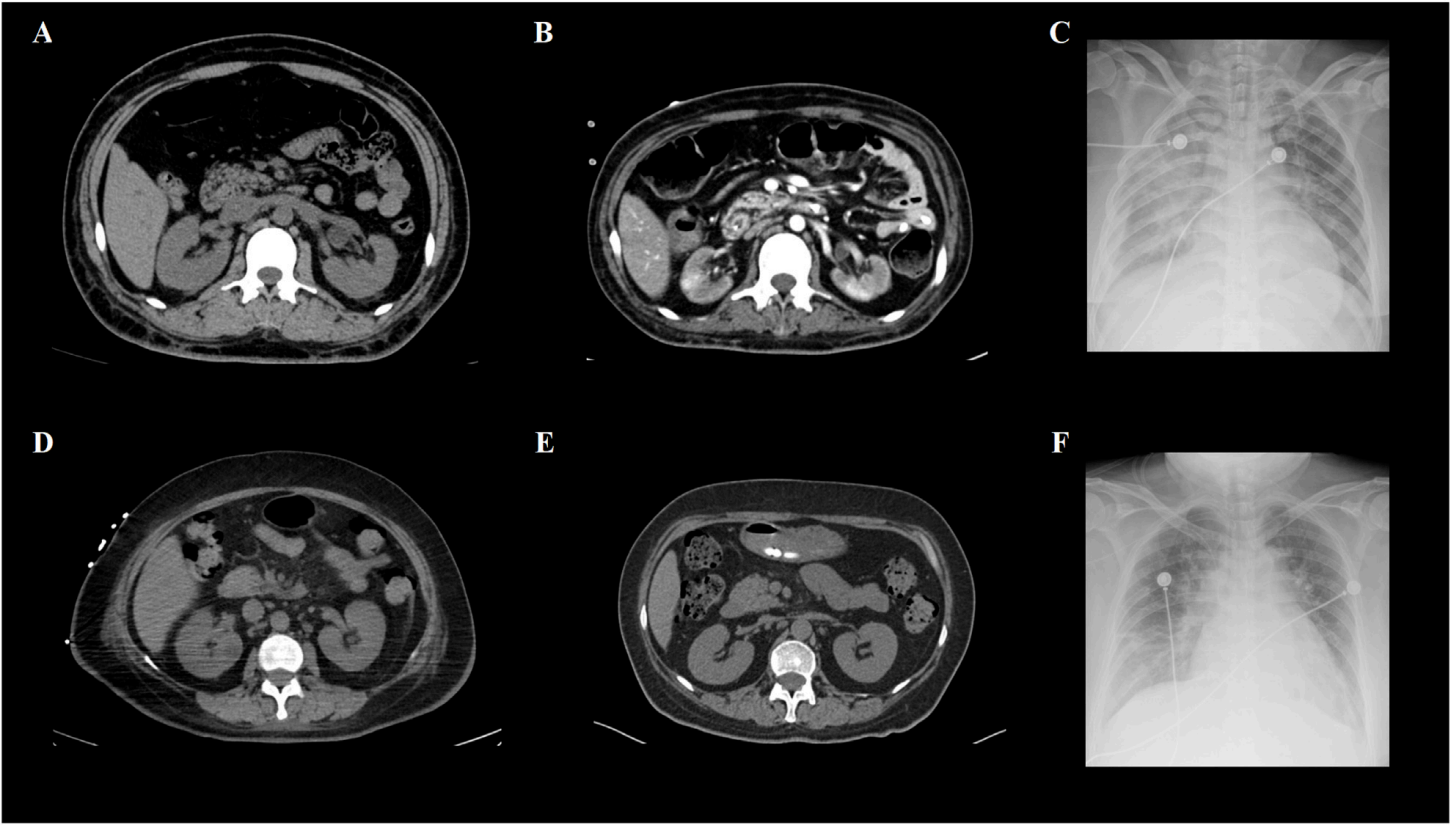
CT and X-ray images of two APL patients with renal failure. **(A)** Abdominal CT of patient #2 revealed kidney swelling and enlargement when DS occurred. **(B)** Patient #2 had persistent renal dysfunction and became dialysis-dependent after achieving complete remission of APL. Enhanced CT imaging 3 months later revealed kidney shrinkage and decreased contrast intake at focal sites, which was consistent with irreversible changes in the renal function. **(C)** Obvious bilateral pulmonary infiltrates caused by DS were demonstrated by the chest X-ray of patient #2. **(D)** Abdominal CT of patient #5 also revealed kidney swelling and enlargement in the DS. **(E)** Unlike patient #2, the renal function of patient #5 was restored to normal after DS was successfully managed. Non-contrast CT imaging 2 months later revealed that both kidneys had normal appearances. **(F)** Similar to patient #2, patient #5 developed pulmonary infiltration, shown as patch opacities on the chest X-ray at the pinnacle of the DS.

**FIGURE 2:**
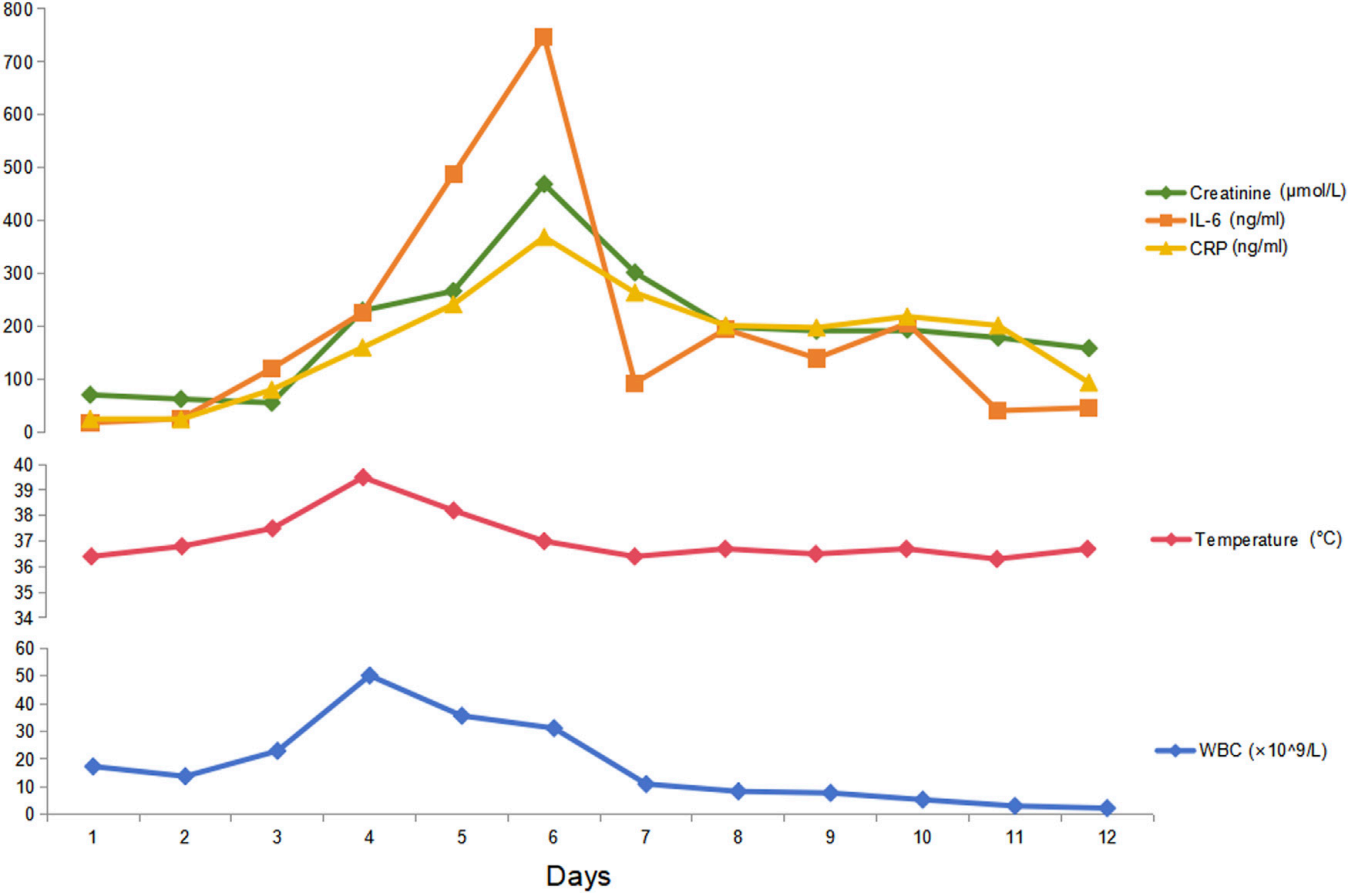
Serial levels of cytokines, renal function parameters, and WBC counts after induction in an APL patient. Patient #2 experienced a rapid increase in WBC, fever, dyspnea, edema, and oliguria after induction therapy with ATRA and ATO. Simultaneous monitoring of her cytokine IL-6 and CRP levels revealed that both IL-6 and CRP increased during the DS process. This patient soon developed renal failure, as shown by anuria, congestive heart failure, and a rapid increase in serum creatinine. Thus, CRRT was initiated together with dexamethasone (20 mg per day until DS was relieved) and daunorubicin (60 mg per day for 3 days). The cytokines peaked on day 6 and started to decrease along with the alleviation of DS symptoms and the normalization of WBC.

A total of 8/26 (30.8%) patients received CRRT due to the development of anuria and/or congestive heart failure. The clinical characteristics of these eight patients are summarized in Table 2. Among them, three were high-risk APL patients, and two had extreme hyperleukocytosis, defined as an initial WBC count exceeding 100 × 10^9^/L. Seven patients experienced rapid and remarkable WBC elevation as a sign of DS. AKI generally develops within 5 days (range, 0–5 days) after induction. Five patients survived for more than 3 months. Nevertheless, three of these five non-ED patients were still dependent on regular hemodialysis without renal function recovery during the follow-up. Among the eight patients who received CRRT, two patients (patients #2 and #5) underwent abdominal computerized tomography (CT) when AKI occurred, and bilateral kidney swelling and slight nephromegaly were found on CT imaging in both patients (Figures 1A, D). In addition, both patients developed pulmonary edema and infiltration on chest X-ray, which indicated severe DS (Figures 1C, F). Moreover, obvious decreases in renal size and hypoperfusion signs were observed on serial CT 3 months later in patient #2, who experienced persistent renal dysfunction and was continuously dependent on dialysis. In contrast, patient #5 had restored normal renal function, and CT imaging 2 months later revealed the disappearance of renal edema with the restoration of the normal kidney size (Figures 1B, E). One patient (patient #2) had simultaneous monitoring of interleukin (IL)-6 and C-reactive protein (CRP) levels during the development of DS. A synchronized change in the WBC and cytokine trends was documented (Figure 2). Among the 18 patients who had AKI but did not require renal replacement therapy in the induction phase, 11/18 (61.1%) patients were demonstrated to have normalized renal function and survived over 3 months of follow-up. Nevertheless, the remaining 7/18 (38.9%) patients either developed chronic kidney disease or experienced ED.

**TABLE 2:** Summary of APL patients who received dialysis during induction treatment.

No	Age/gender	WBC (×10^9^/L)	PT (s)	APTT (s)	TT (s)	Fib (g/L)	D-dimer (mg/L)	sCr (μmol/L)	LDH (U/L)	APL risk	Initial ATRA dose (mg)	WBCmax (×10^9^/L)	Development of renal dysfunction since induction (days)	Dialysis-dependent at endpoint	Outcome	OS (months)
1	15/M	8.17	16.1	28.3	23.0	0.62	12.85	376	1766	Intermediate	40	23.64	0	Yes	Death	1.1
2	34/F	17.36	15.3	23.6	18.4	0.85	15.85	71	465	High	40	50.22	3	Yes	Survival	4.2
3	58/F	0.47	11.1	26.3	16.6	2.70	5.88	47	134	Intermediate	40	7.69	2	No	Survival	28.2
4	30/F	1.68	21.4	64.4	20.1	0.81	38.00	88	242	Intermediate	40	35.53	5	Yes	Death	28.6
5	34/M	4.84	13.3	23.9	16.4	1.63	4.04	90	152	Intermediate	30	19.8	1	No	Survival	121.4
6	57/F	140.86	15.2	24.0	25.1	0.50	25.64	49	2,302	High	20	169.16	2	Yes	Death	12.3
7	64/M	120.19	19.5	27.8	19.4	0.66	38.00	186	1,292	High	20	120.19	0	Yes	Death	0.7
8	52/F	10.59	17.0	25.5	17.9	0.65	38.00	148	460	High	80	94.52	0	Yes	Death	0.2

Abbreviations: M, male; F, female; WBC, white blood cell; PT, prothrombin time; APTT, activated partial thromboplastin time; TT, thrombin time; Fib, fibrinogen; sCr, serum creatinine; LDH, lactate dehydrogenase; APL, acute promyelocytic leukemia; ATRA, all-trans retinoic acid; WBCmax, peak white blood cell count; OS, overall survival.

### 3.3 Survival and risk factors

**FIGURE 3:**
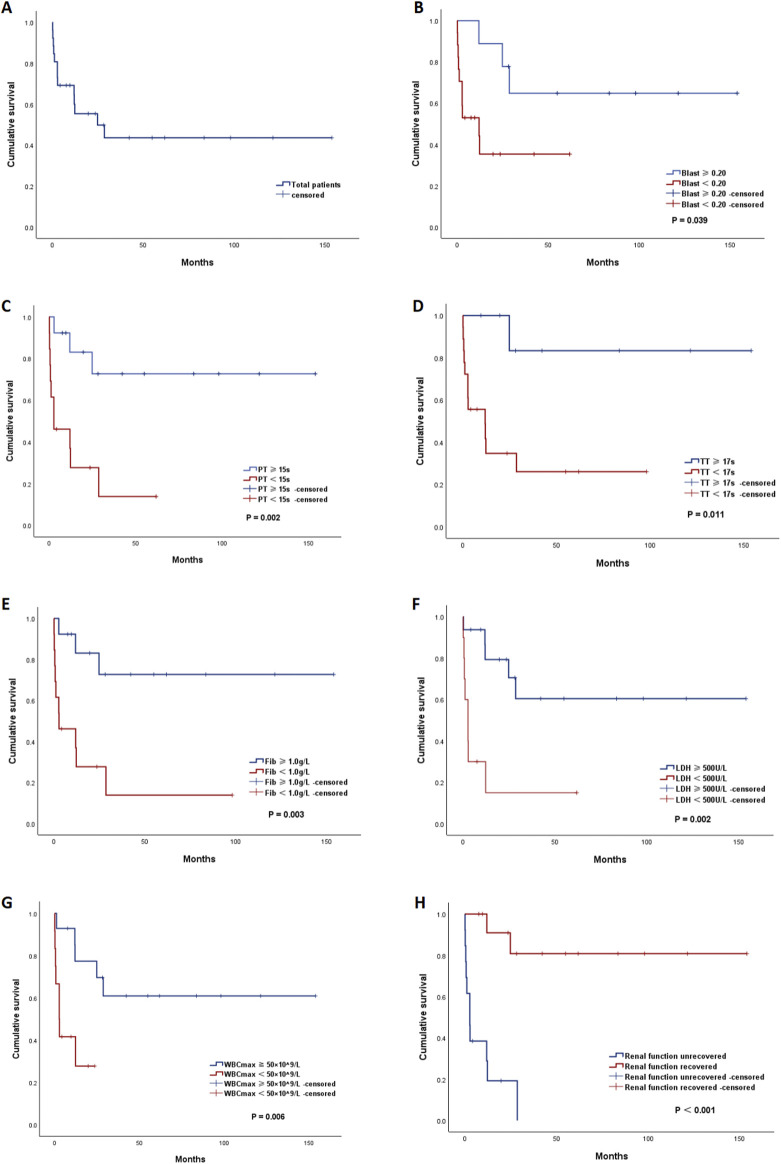
Kaplan‒Meier (KM) curves of overall survival. **(A)** KM curve of the total patients. KM curves of all APL patients stratified by different risk factors: **(B)** peripheral blood blast percentage (blast percentage ≥ 0.20 vs. blast percentage<0.20), **(C)** prothrombin time (PT) (PT ≥ 15 s vs. PT < 15 s), **(D)** thrombin time (TT) (TT ≥ 17 s vs. TT < 17 s), **(E)** fibrinogen level (Fib) (Fib ≥ 1.0 g/L vs. Fib < 1.0 g/L), **(F)** lactate dehydrogenase (LDH) (LDH ≥ 500 U/L vs. LDH < 500 U/L), **(G)** peak white blood cell count (WBCmax) (WBCmax≥50 × 10^9^/L vs. WBCmax<50 × 10^9^/L), and **(H)** persistence of renal dysfunction (yes vs. no).

After a median follow-up of 54.9 months (range, 0.1–153.8 months), the median OS of all patients was 24.8 months (95% confidence interval [CI]: 0∼53.5) (Figure 3A). The estimated 2-year OS rate was 42%. Eight patients died within 3 months after diagnosis, resulting in an ED rate of 30.8%. The direct causes of ED were sepsis (3/8), intracranial hemorrhage (2/8), gastrointestinal bleeding (1/8), myocardial injury (1/8), and a large area of cerebral infarction (1/8).

To identify the risk factors associated with adverse outcomes, we evaluated the clinical characteristics, including demographic features, laboratory parameters at diagnosis, clinical manifestations, and treatment and renal outcomes, between the non-ED and ED groups, and these characteristics were compared between the total survival group and the death group (Table 3). Compared with non-ED patients, ED patients had greater WBC counts (×10^9^/L) (80.81 vs. 4.15, p = 0.005), blast percentages in the peripheral blood (0.88 vs. 0.22, p = 0.001), prothrombin times (PTs) (seconds, s) (16.8 vs. 13.2, p = 0.004) and thrombin times (TTs) (20.0 vs. 17.6, p = 0.026), D-dimer levels (mg/L) (36.00 vs. 11.59, p = 0.042), fibrinogen levels (g/L) (0.49 vs. 1.68, p = 0.001), and lactate dehydrogenase (LDH) levels (U/L) (1,470 vs. 308, p = 0.001) at diagnosis. Moreover, ED patients had higher peak WBC counts (WBCmax) (×10^9^/L) (134.78 vs. 36.65, p = 0.001) and a greater incidence of persistent renal dysfunction after induction therapy (100% vs. 27.8%, p < 0.001). Compared with the total survival group, the total death group had a greater blast percentage in the peripheral blood (0.82 vs. 0.28, p = 0.019), longer PT(s) (16.6 vs. 13.1, p = 0.009) and TT(s) (20.1 vs. 16.8, p = 0.012), lower fibrinogen (g/L) (0.78 vs. 1.75, p = 0.003), and higher LDH (U/L) (1,292 vs. 313, p = 0.029).

**TABLE 3:** Potential factors related to death.

Factor	Survival at 3 months (n = 18)	Death at 3 months (n = 8)	P value	Survival (n = 13)	Death (n = 13)	*p*-value
	N (%)/Median (range)	N (%)/Median (range)		N (%)/median (range)	N (%)/median (range)	
Male gender	10 (55.6)	5 (62.5)	0.543	8 (61.5)	6 (46.2)	0.500
Age (years)	54.5 (27–70)	35.0 (15–64)	0.031	52.0 (27–69)	43.0 (15–70)	0.538
Duration of symptoms before admission (days)	5 (1–30)	5 (2–19)	0.780	6 (1–30)	4 (2–29)	0.840
Laboratory parameter at diagnosis						
Hb (g/L)	71 (30–154)	67 (28–120)	0.539	69 (30–154)	75 (28–120)	0.909
Plt count (×10^9^/L)	23 (7–72)	29 (3–44)	0.611	22 (7–72)	32 (3–59)	0.606
WBC count (×10^9^/L)	4.15 (0.35–140.86)	80.81 (8.17–152.26)	0.005	5.86 (0.35–57.80)	63.42 (0.53–152.26)	0.091
Peripheral blood blast percentage	0.22 (0–0.98)	0.88 (0.79–0.92)	0.001^*^	0.28 (0–0.90)	0.82 (0–0.98)	0.019^*^
PT(s)	13.2 (10.5–48.2)	16.8 (14.8–23.8)	0.004^*^	13.1 (11.1–17.4)	16.6 (10.5–48.2)	0.009
APTT(s)	26.3 (22.6–64.4)	29.8 (25.5–51.7)	0.177	26.3 (22.6–36.5)	28.3 (23.6–64.4)	0.204
TT(s)	17.6 (15–26)	20.0 (17.6–26.3)	0.026^*^	16.8 (15.4–23.6)	20.1 (16.3–26.3)	0.012^*^
D-dimer (mg/L)	11.59 (4.04–38)	36.00 (9.49–38)	0.042^*^	8.97 (4.04–38.00)	31.24 (8.49–38.00)	0.064
Fib (g/L)	1.68 (0.50–4.39)	0.49 (0.50–1.14)	0.001^*^	1.75 (0.59–4.35)	0.78 (0.50–2.60)	0.003^*^
LDH(U/L)	308 (134–2,304)	1,470 (460–2,977)	0.001^*^	313 (134–1,329)	1,292 (160–2,799)	0.029^*^
Creatinine (μmol/L)	89 (32–178)	156 (66–378)	0.063	83 (32–178)	124 (49–376)	0.069
Uric acid (mmol/L)	330.5 (140–611)	426.5 (288–549)	0.346	340 (140–611)	410.7 (251–547)	0.259
High-risk APL	7 (38.9)	7 (87.5)	0.280	6 (46.2)	8 (61.5)	0.348
Manifestation and complication at diagnosis						
Mucocutaneous bleeding	8 (44.4)	6 (75.0)	0.155	7 (53.8)	7 (53.8)	0.652
Pulmonary infiltration on CT	7 (38.9)	4 (50.0)	0.549	4 (30.8)	7 (53.8)	0.214
Intracranial bleeding on CT	1 (5.6)	2 (12.5)	0.529	1 (7.7)	1 (7.7)	0.760
Gastrointestinal bleeding	3 (16.7)	3 (37.5)	0.249	3 (23.1)	3 (23.1)	0.678
Respiratory failure	1 (5.6)	3 (25.0)	0.215	1 (7.7%)	2 (15.4)	0.500
Presence of renal dysfunction before induction	8 (44.4)	5 (65.5)	0.336	5 (38.5)	8 (61.5)	0.217
Treatment and outcome						
Initial ATRA doses (mg)	35 (15–40)	20 (10–80)	0.238	40 (10–40)	20 (10–80)	0.287
WBCmax (×10^9^/L)	36.65 (7.69–169.16)	134.78 (23.64–203.92)	0.001^*^	28.70 (6.67–60.00)	120.19 (14.48–203.92)	0.002^*^
Development of renal dysfunction after induction (days)	1.5 (0–16)	0 (0–12)	0.338	2 (0–16)	0 (0–12)	0.204
Dialysis	5 (27.8)	3 (37.5)	0.478	3 (23.1)	5 (38.5)	0.336
Persistence of renal dysfunction	5 (27.8)	8 (100)	<0.001^*^	2 (15.4)	11 (84.6)	<0.001^*^

Abbreviations: Hb, hemoglobin; Plt, platelet; WBC, white blood cell; PT, prothrombin time; APTT, activated partial thromboplastin time; TT, thrombin time; Fib, fibrinogen; LDH, lactate dehydrogenase; APL, acute promyelocytic leukemia; ATRA, all-trans retinoic acid; WBCmax, peak white blood cell count.

*p<0.05.

Survival analysis via Kaplan–Meier methods (Table 4) revealed that patients with a blast percentage ≥0.20, PT ≥ 15 s, TT ≥ 17 s, fibrinogen <1.0 g/L, LDH ≥2 × UNL (500 U/L), WBCmax ≥50 × 10^9^/L, and persistent renal dysfunction had significantly inferior survival (Figures 3B–H). In the multivariate analysis, persistent renal dysfunction was found to be an independent risk factor for OS (hazard ratio [HR] = 0.101, 95% CI 0.012–0.888).

**TABLE 4:** Survival of different risk groups.

Risk factor		N	Univariate analysis		Multivariate analysis	
			Estimated median OS	*p-*value	HR (95% CI)	*p-*value
Peripheral blood blast percentage	≥0.20	17	2.8	0.039	2.917 (0.181–46.925)	0.450
	<0.20	9	NR			
PT	≥15 s	13	2.9	0.002	1.026 (0.190–5.556)	0.976
	<15 s	13	NR			
TT	≥17 s	18	11.9	0.011	2.372 (0.105–53.598)	0.587
	<17 s	8	NR			
Fib	<1.0 g/L	13	2.8	0.003	1.370 (0.238–7.882)	0.724
	≥1.0 g/L	13	NR			
LDH	≥2×UNL	10	2.7	0.002	1.566 (0.247–9.944)	0.634
	<2×UNL	16	NR			
WBCmax	≥50 × 10^9^/L	12	2.7	0.006	0.707 (0.097–5.166)	0.733
	<50 × 10^9^/L	14	NR			
Renal function	unrecovered	13	2.7	<0.001	0.101 (0.012–0.888)	0.039
	recovered	13	NR			

Abbreviations: NR, not reached; PT, prothrombin time; TT, thrombin time; Fib, fibrinogen; LDH, lactate dehydrogenase; UNL, upper normal limit; WBCmax, peak white blood cell count; OS, overall survival.

## 4 Discussion

In our study, we identified 26 AKI patients among 572 APL patients treated with an ATRA plus ATO-based dual-induction regimen over a 12-year span. On the basis of data from the PETHEMA study, which included 183 DS cases following the AIDA regimen, patients with four or more DS signs or symptoms were classified as having “severe” DS. In this study, 90 out of 183 DS cases were categorized as severe (Montesinos et al., 2009). Moreover, the incidence of renal failure was much greater in the severe DS group than in the moderate DS group (46% vs. 9%, P < 0.001). However, the GIMEMA group, which also used the AIDA regimen, reported an incredibly low incidence of DS (2.5%, 6 of 240 patients) (Mandelli et al., 1997). Owing to the use of different diagnostic criteria for DS and AKI and the heterogeneous induction regimens employed in various studies, the actual incidence of DS-related AKI is unclear. In our study, three out of the eight patients who needed renal replacement therapy died within 3 months. Among the five non-ED patients, three were still dependent on hemodialysis without recovery of renal function. Thus, irreversible renal failure seems to be a rare but notable complication of severe DS, which has been previously discussed only in sporadic case reports (Flombaum et al., 1996; Frankel et al., 1992).

The etiopathogenic mechanisms of DS as a life-threatening complication in APL patients are complex and remain incompletely understood. Moreover, the exact mechanism behind this irreversibility of renal dysfunction is not well-understood. The hypothesis is that DS behaves like a systemic inflammatory response system and capillary leak syndrome (Iyer et al., 2023). ATRA targets the ATRA receptor and induces terminal differentiation of APL blasts. The differentiation of APL cells can stimulate chemokine production in target organs. These chemokines serve as chemoattractants for other inflammatory cells, which further exacerbates the hyperinflammatory state (Luesink et al., 2009b). Thus, AKI in APL patients could result from a therapeutic intervention-induced cytokine storm syndrome (CSS), similar to the hyperinflammation status following immunotherapy, such as monoclonal antibody administration and chimeric antigen receptor (CAR) T-cell therapy (Karki and Kanneganti, 2021). CSS is triggered by the activation of bystander cells, particularly myeloid cells, in the tumor environment. Subsequent inflammatory cell death and tumor lysis induce CSS through the release of inflammatory cytokines (Lee et al., 2014). Pro-inflammatory cytokines, including IL-6, IL-1, and IFN-γ, are consistently elevated in the serum of patients with CSS (Liu et al., 2020). Another contributing factor is coagulopathy, which is extremely common in cytokine storm-related conditions. In addition to DIC, coagulopathy in CSS is a thrombotic or hypercoagulable condition due to the interplay between inflammation and clotting cascades. Like in COVID-19 infection, the systemic inflammatory state can trigger coagulation cascades, which, in turn, activate the production and release of pro-inflammatory mediators via positive feedback mechanisms (Savla et al., 2021). In the setting of DS, leukostasis secondary to hyperleukocytosis could further aggravate microthrombosis and tissue ischemia of the target organ. The rapid correction of the hypocoagulable status after induction therapy in APL patients makes microthrombosis more troublesome in capillary-rich organs such as the kidney. Moreover, lower oxygen delivery to the kidney, directly or indirectly caused by interstitial edema, vasoconstriction, circulation collapse, respiratory failure, and septic complications, could also contribute to the development of AKI (Ahmadian et al., 2021). Thus, if the cytokine storm cannot be harnessed in time, inflammation-related tissue injury and cellular necrosis rapidly develop widespread microthrombosis, and hypoperfusion might work together to explain permanent kidney injury. Unfortunately, owing to the obvious coagulopathy and critical condition of APL patients during the induction period, renal biopsy is almost impossible. However, some postmortem studies of patients with DS-related renal failure have shown diffuse neutrophilic and leukemic infiltration in multiple organs, including the kidney (Flombaum et al., 1996; Kakkar et al., 2002)^.^ The infiltration of leukemic cells is suggested to be mediated by the release of a variety of cytokines by differentiating blast cells and the altered adhesion properties of the blast cells after being primed by ATRA (Woods and Norsworthy, 2023). ATRA treatment can increase the expression of adhesion molecules such as CD11b, CD18, and ICAM-1, which increases the adhesion of myeloid cells to endothelial cells, facilitating their migration into tissues (Mohammadzadeh et al., 2021). The presence of enlarged kidneys shown by ultrasonography during the period of leukocytosis, the disappearance of nephromegaly after recovery, and the prompt response to steroids and chemotherapy in previous reports were highly suggestive of leukemic infiltration of the kidneys under the inflammatory storm as the cause of renal failure (Montesinos and Sanz, 2011; Frankel et al., 1992). The morphological changes in the kidneys observed via serial CT imaging in our study also reflected this pathophysiological process. Moreover, in our study, two of the five non-ED patients did not receive dialysis, and the peak WBC counts of the two survivors were 7.69 and 19.8 × 10^9^/L, respectively, which were much lower than those of patients who had ED- or dialysis-dependent survival. Thus, when DS occurs, the rate at which the WBC count increases and the peak WBC count during the differentiation process could be associated with the severity of leukemic infiltration in affected organs and with unfavorable outcomes in APL patients. Thus, AKI in DS patients is considered a multifactorial event.

In the future, a deeper mechanistic understanding of the role of the “cytokine storm” in the pathogenesis of AKI secondary to DS is crucial for devising targeted therapy. Recently, several novel anti-cytokine therapies, such as anti-IL-6 receptor antibodies and anti-IFN-γ antibodies, have achieved success in hemophagocytic lymphohistiocytosis and CAR-T-cell therapy-induced CSS (Fajgenbaum and June 2020). Thus, in addition to corticosteroid and cytoreductive chemotherapy, these novel agents could be explored for more efficient control of DS and salvage of renal function. Moreover, anticoagulation therapy, such as low-dose heparin, might help prevent catastrophic microthrombosis in APL patients.

The concomitant administration of cytoreductive chemotherapy and the use of high-dose dexamethasone at the onset of DS symptoms appear to have significantly reduced DS-related mortality to 1% (Montesinos et al., 2009; Tallman et al., 2000; Lo-Coco et al., 2013). In the PETHEMA series, DS-associated mortality was 11% in patients with severe DS, whereas no deaths resulted from moderate DS (Montesinos et al., 2009). In our study, APL patients with DS-related AKI still had dismal outcomes. Although hemodialysis or CRRT is often promptly available in our hospital when refractory renal failure and fluid overload occur, eight patients (30.2%) suffered from ED due to sepsis, intractable bleeding, and DIC-related thrombosis of the heart or brain, with renal failure not being the direct cause of death. The higher WBC count at diagnosis and peak WBC count after induction, blast percentage, and LDH found in ED patients reflected the impact of tumor burden on the risk of DC, whereas the lower DIC indices at diagnosis suggested that hemorrhage and thrombosis, the two edges of the same sword of DIC, also play a role in its prognostication. Finally, the estimated 2-year OS rate in this study was only 42%. Survival analysis also confirmed that patients with heavier tumor burdens reflected by higher blast percentages, LDH levels, and peak WBC counts, together with worse DIC parameters, had much poorer long-term survival. In addition, the persistence of renal dysfunction was significantly associated with a higher ED rate and poorer survival. Thus, renal failure seems to be a more detrimental event in DS patients and is associated with the worst outcome for APL patients in all risk groups. The management of acute renal failure as a consequence of DS in APL patients is still an unmet clinical need.

Under the current framework of APL treatment, there is no consensus on the strength of the cytoreductive chemotherapy we should apply to APL patients with severe DS (Iyer et al., 2023; Kayser et al., 2018). As hyperleukocytosis is associated with the development of DS and ED (Woods and Norsworthy, 2023), in addition to the use of high-dose dexamethasone and the discontinuation of differentiating agents, we believe that adequate cytoreductive chemotherapy should be instantaneously initiated when the WBC count is rapidly increasing and signs of renal dysfunction start to occur during the induction period so that infiltrating leukemic cells in the kidneys can be exterminated more quickly and irreversible organ injury can be avoided. Moreover, aggressive support care should be guaranteed, including close DIC monitoring and correction, by administering cryoprecipitate and other coagulation factor concentrates instead of fresh-frozen plasma to avoid further volume overload (Yilmaz et al., 2021; Gao et al., 2019). The availability of renal replacement therapy and mechanical ventilation, along with a strong multidisciplinary team and the joint efforts of nephrologists and physicians from the intensive care unit (ICU), is also crucial for the successful management of this special group of patients. Issues such as fluid balance, ventilation strategies for better control of pulmonary edema, optimized anticoagulation in CRRT, and salvage of sepsis are all dependent on the strength of a multidisciplinary team and adequate communication between hematologists and physicians from nephrology and the ICU. Another issue is the safety and dosage of ATO in acute renal failure patients with APL. Although there are reports that ATO can be safely and effectively used to treat APL patients undergoing hemodialysis (Jen et al., 2024; Perreault et al., 2016), a recent study investigating the risk factors for AKI in APL patients revealed that an ATO dose exceeding 15 mg per day was a significant predictor of clinically significant AKI (odds ratio = 1.91, 95% CI = 1.19–3.07, p = 0.007) (Fajgenbaum and June 2020). Thus, considering that high-dose ATO may be associated with significant nephrotoxicity, we recommend that the ATO dose should be capped at 15 mg to minimize its toxicity. Finally, for patients who develop irreversible renal injury and evolve into end-stage renal disease after achieving complete remission, kidney transplantation, which is better suited than dialysis, seems to be the only long-term solution (Yoon et al., 2019). As APL has a remarkably favorable prognosis, kidney transplantation can be considered without a lengthy period of waiting.

Limitations to this study include a relatively small population sample and a lack of detailed information for certain patients due to the long span and retrospective nature and single-center design of the study. Heterogeneity in patient management and possible selection bias as a real-world study may also introduce certain confounders. No adjustments for multiple comparisons were made in the *t*-test, given the small sample size. Similarly, the statistical strength of the Cox regression model may be limited. Thus, in the future, multicenter prospective studies with more patients are needed to validate the results of this research.

## 5 Conclusion

This retrospective study revealed that AKI is a rare but severe complication of DS in APL patients treated with ATRA + ATO dual induction. It is associated with a high ED rate and dismal long-term survival. Among patients in this special APL group, those with a greater tumor burden, rapidly increasing WBC count, worse coagulation parameters, and persistent renal dysfunction had significantly poorer outcomes. In the dual-induction era, even with adequate cytoreductive chemotherapy, dexamethasone treatment, and instantaneous CRRT support, some patients still develop irreversible renal failure and become dialysis-dependent after achieving remission from APL. The management of AKI as a consequence of DS in APL patients is a clinical challenge, and a deeper understanding of its pathogenesis, along with multidisciplinary efforts, is needed.

## Data Availability

The raw data supporting the conclusions of this article will be made available by the authors, without undue reservation.
